# Lentiviral Vector Mediated Claudin1 Silencing Inhibits Epithelial to Mesenchymal Transition in Breast Cancer Cells

**DOI:** 10.3390/v7062755

**Published:** 2015-06-10

**Authors:** Xianqi Zhao, Yanan Zou, Qingqing Gu, Guannan Zhao, Horace Gray, Lawrence M. Pfeffer, Junming Yue

**Affiliations:** 1 Department of Medicine, Harbin Medical University, Harbin 150086, China; E-Mails: ynzou@sina.com (X.Z.); yzou4@uthsc.edu (Y.Z.); 2 Department of Pathology and Laboratory Medicine, University of Tennessee Health Science Center, Memphis, TN 38163, USA; E-Mails: qgu4@uthsc.edu (Q.G.); gzhao4@uthsc.edu (G.Z.); horacegray1991@yahoo.com (H.G.); lpfeffer@uthsc.edu (L.M.P.); 3 Center for Cancer Research, University of Tennessee Health Science Center, 19 S. Manassas St., Rm. 266, Memphis, TN 38163, USA

**Keywords:** CLDN1, epithelial to mesenchymal transition, breast cancer

## Abstract

Breast cancer has a high incidence and mortality rate worldwide. Several viral vectors including lentiviral, adenoviral and adeno-associated viral vectors have been used in gene therapy for various forms of human cancer, and have shown promising effects in controlling tumor development. Claudin1 (CLDN1) is a member of the tetraspan transmembrane protein family that plays a major role in tight junctions and is associated with tumor metastasis. However, the role of CLDN1 in breast cancer is largely unexplored. In this study, we tested the therapeutic potential of silencing CLDN1 expression in two breast cancer (MDA-MB-231 and MCF7) cell lines using lentiviral vector mediated RNA interference. We found that a CLDN1 short hairpin (shRNA) construct efficiently silenced CLDN1 expression in both breast cancer cell lines, and CLDN1 knockdown resulted in reduced cell proliferation, survival, migration and invasion. Furthermore, silencing CLDN1 inhibited epithelial to mesenchymal transition (EMT) by upregulating the epithelial cell marker, E-cadherin, and downregulating mesenchymal markers, smooth muscle cell alpha-actin (SMA) and Snai2. Our data demonstrated that lentiviral vector mediated CLDN1 RNA interference has great potential in breast cancer gene therapy by inhibiting EMT and controlling tumor cell growth.

## 1. Introduction

Breast cancer is the most common cancer in women and the leading cause of cancer death. There are over 40,000 deaths reported annually in the US, although the mortality rate is declining yearly [1]. A reason for the high mortality rate is inefficient chemotherapeutic drug delivery. Several approaches have been used to improve the gene delivery system including using lipids, ligands, nanoparticles, polymers and viral vectors as delivery agents [2]. Viral vectors, including adenoviral, adeno-associated, retroviral and lentiviral vectors, have high transduction efficiency in experimental animal models and have shown promise in clinical trials for treating a variety of human cancers [3,4,5,6,7,8]. Viral vector mediated RNA interference has been widely used to silence the expression of various target genes. In particular, silencing oncogene expression in cancer cells has therapeutic potential. Gene expression has been silenced in several studies by replacing the mature miRNA sequences of miR-21 and miR-30 with target gene sequences [9,10]. Lentiviral vectors have been used widely for gene delivery including RNA interference. Lentiviral vectors have several important advantages over other viral vector systems in cancer therapy, including long term expression, high transduction efficiency, less antitumor immunity [11]. Furthermore, the most widely used lentiviral vector presently is a third generation vector modified by deleting viral promoter and separating viral structural genes into three packaging plasmids, which significantly reduces the possibility of generating replication competent viruses.

Claudins are a family of 25 transmembrane proteins [12], but their role in human cancers is not fully understood. CLDN1 has been shown to function as an oncogene or tumor suppressor depending on the specific cellular context. CLDN1 has oncogenic activity in colon cancer [13], bladder cancer [14], lung cancer [15], gastric cancer [16], ovarian cancer [17], and melanoma [18], but functions as a tumor suppressor in colorectal cancer (CRC), and its expression was inversely correlated with prognosis and overall survival [19]. CLDN1 was differentially expressed during pregnancy, lactation, and involution in the normal mammary gland and mammary tumors [20]. However, the role of CLDN1 in breast cancer is largely unclear. Decreased CLDN1 expression is correlated with lower disease-free survival and lymph node metastasis [21]. In triple-negative breast cancer (TNBC), the low expression of CLDN1-correlates with a high risk of recurrence and death [22]. However, high expression levels of CLDN1 were found in the aggressive basal-like breast cancer subtypes. Silencing CLDN1 in the BT-20 breast cancer cell line inhibited cell migration and EMT [23]. CLDN1 was shown to have an antiapoptotic effect in MCF7 breast cancer cells [24]. Therefore, further investigation is required to determine whether CLDN1 expression is correlated with the development of different subtypes of breast cancer, and understand the molecular mechanisms underlying the role of CLDN1 in metastasis.

The cellular phenotypic switch from epithelial to mesenchymal cell transition (EMT) is associated with tumor initiation, progression, metastasis, and chemoresistance [25,26,27,28,29]. The expression of EMT marker genes, including Snai1 and 2, Zeb 1 and 2, Twist1 and 2, vimentin, and E-cadherin, was altered during this process [30,31,32]. In this study, we silenced expression of CLDN1 in breast cancer MDA-MB-231 and MCF7 cells using highly efficient lentiviral vector mediated CLDN1 RNA interference and found that silencing CLDN1 inhibits cell proliferation, migration, and invasion by inhibiting EMT in breast cancer cell lines.

## 2. Materials and Methods

### 2.1. Cell Culture

The breast cancer cell lines, MDA-MB-231 and MCF7, were obtained from ATCC and cultured in Dulbecco’s Modified Eagle Medium (DMEM) supplemented with 10% FBS (Hyclone; Logan, UT, USA), 100 U/mL penicillin, and 100 μg/mL streptomycin (Invitrogen; Carlsbad, CA, USA). HEK293 FT cells were cultured in DMEM media with 10% FBS, 100 U/mL penicillin, 100 μg/mL streptomycin, 1% glutamine, 1% nonessential amino acid, and 1 μg/mL geneticin. Cell morphology was examined under light microscopy and imaged.

### 2.2. Lentiviral Vector Production and Transduction

Lentiviral CLDN1 shRNA vectors (TRCN0000117332, TRCN0000117334) for CLDN1 knockdown were purchased from Dharmacon (Lafayette, CO, USA). Lentiviral scramble control (SC) shRNA (#1864) was purchased from Addgene (Cambridge, MA, USA). All lentiviral vectors were packaged in HEK293FT cells and produced as described previously [9]. CLDN1 shRNA stable cell lines were established by transducing MDA-MB-231 and MCF7 cells with purified virus, and stable pools of cells were selected with 5 μg/mL puromycin.

### 2.3. Cell Colony Formation Assay

MDA-MB-231 and MCF7 cells transduced with lentiviral CLDN1 shRNAs and scramble control vectors were plated at 200 cells/well in triplicate into 6-well plates and then stained with 0.1% Crystal Violet following after incubation for two weeks, and cell colonies were counted as described previously [33].

### 2.4. MTT Assay

Cell proliferation was examined using the MTT assay kit purchased from ATCC (Manassas, VA, USA) following the manufacturer’s instructions. In brief, breast cancer cells were plated at 8000 cells/well in 96-well plates. At various time points, 10 µL of MTT reagent (10 mg/mL) was added to each well and incubated for ~4 h. The reaction was terminated by adding 100 µL of lysis reagent and incubated at 22 °C in the dark for 2 h. The absorbance was measured at 570 nm wavelength on a Bio-Rad plate reader (Bio-Rad Laboratories, Hercules, CA, USA).

### 2.5. Cell Migration Assay

Cell migration was examined using wound healing and transwell plates as previously described [34]. The wound healing assay was performed by scratching the monolayer of cells and then measuring the migration area at 0 and 24 h. Migration index was calculated using the formula: (wound area at 0 h-wound area at 24 h)/wound area at 0 h × 100%. The transwell migration assay was performed using a modified chamber system from BD Falcon™ (San Jose, CA, USA). These chambers were inserted into a 24-well plate with 3 × 10^4^ of cells in 300 µL serum-free DMEM added to the upper chamber. DMEM (supplemented with 10% of FBS) was added into the lower chamber of each well as a chemoattractant, and cells were incubated for 24 h. The medium and non-migrated cells in the upper chamber were removed, whereas the migrated cells on the lower side of the membranes were fixed with methanol and stained with 0.1% Crystal Violet. Pictures were taken at 10× magnification. Transmigrated cells in at least three different fields were counted.

### 2.6. Cell Invasion Assay

MDA-MB-231 and MCF7 cells (5 × 10^5^ per well) transduced with CLDN1 and SC lentiviral vectors were seeded in serum-free DMEM onto inserts precoated with Matrigel (BD BioCoat™, BD BioSciences, San Jose, CA, USA). DMEM supplemented with 10% FBS was added to the bottom chamber and 24 h later, the transwell inserts were stained using 4 µg/mL of Calcein AM (Life Technologies; Grand Island, NY, USA) at 37 °C for 1 h. The fluorescence intensity was measured using the BioTek Synergy™ plate reader (Winooski, VT, USA) at excitation and emission wavelengths of 485 nm and 528 nm, respectively. The invasion rate was calculated by the fluorescence of invading cells in CLDN1 shRNA knockdown cells/control cells ×100.

### 2.7. Western Blot

Breast cancer cells were collected in RIPA buffer (Thermo Scientific; Rockford, IL, USA) containing 1% Halt Proteinase Inhibitor Cocktail (Thermo Scientific). Protein lysates (40 µg/lane) were loaded onto 10% SDS-PAGE gels and transferred onto nitrocellulose membranes. The membranes were blocked with 5% non-fat milk for 1 h and incubated with primary antibodies (1:1000 dilution) against SMA, glyceraldehyde 3-phosphate dehydrogenase (GAPDH; Sigma; St. Louis, MO, USA), vimentin, Claudin1, E-cadherin and Snai2 (1:1000, Cell Signaling, Danvers, MA, USA).

### 2.8. TCGA Database Query

To examine the association of the CLDNs family with breast cancer, we queried the TCGA Database (https://tcga-data.nci.nih.gov/tcga/tcgaHome2.jsp). The data set was filtered for samples on CLDNs and clinical data.

### 2.9. Statistical Analysis

Data from at least two independent experiments performed in triplicate was subjected to Student’s *t-*tests, and presented as means ± S.D. *p* < 0.05 was considered significant.

## 3. Results

### 3.1. Clinical Significance of CLDN1 Expression in Breast Cancer

**Figure 1:**
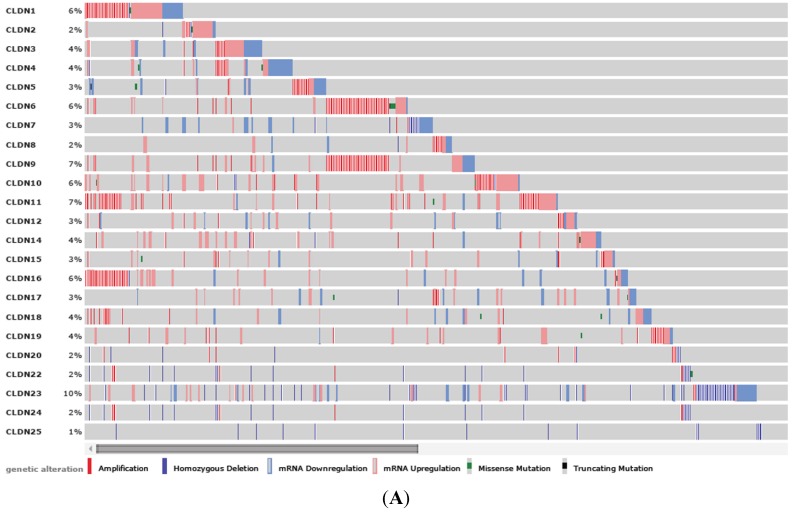

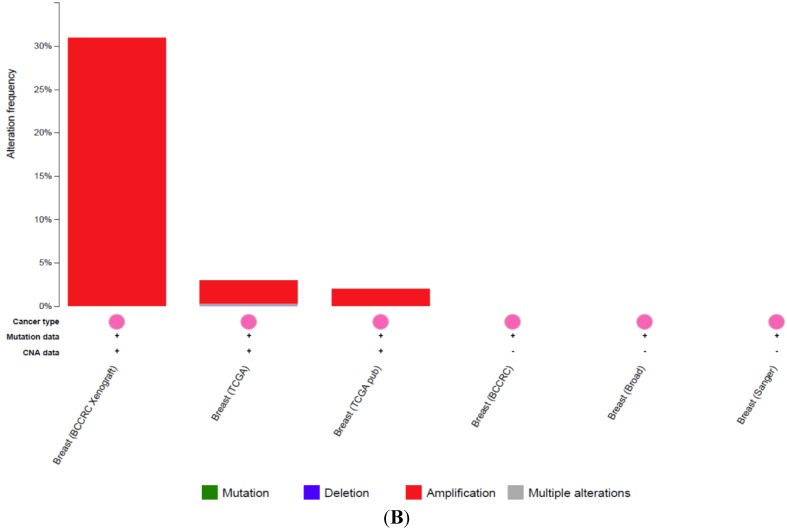
Alterations of claudin (CLDN) family members in breast cancer database. (**A**) Alteration pattern (amplification, upregulation, downregulation, mutation and homozygous deletion) of CLDN family members including CLDN1 to CLDN25 except CLDN13 and 21; (**B**) CLDN1 alterations in invasive breast carcinoma collected from six different datasets.

To determine the clinical significance of claudins in breast cancer, we first analyzed gene alteration frequencies including amplification, upregulation, downregulation, mutation and homozygous deletion. All CLDNs except CLDN13 and CLDN21 were altered in 483 of the 1104 (43.8%) breast cancer cases in the (TCGA) dataset. It is important to note that there are no data available for CLDN13 and CLDN21 from the database. The most altered genes in Claudin family are CLDN1 (6%), CLDN6 (6%), CLDN9 (7%), CLDN10 (6%), CLDN11 (7%), CLDN16 (6%) and CLDN23 (10%), all of them showed amplification and upregulation except that for CLDN23, homozygous deletion was found in the majority of altered samples (Figure 1A). We further examined the alteration of CLDN1 from six different datasets including breast invasive carcinoma (Broad, Sanger, TCGA, British Columbia, Nature, 2012 ) and breast cancer patient xenograft (Nature, 2014) and found that CLDN1 was amplified among samples collected in TGCA and patient xenograft dataset (Figure 1B). We also analyzed the correlation of CLDN1 alterations with the patient survival.

### 3.2. Silencing CLDN1 Inhibits Breast Cancer Cell Proliferation

**Figure 2:**
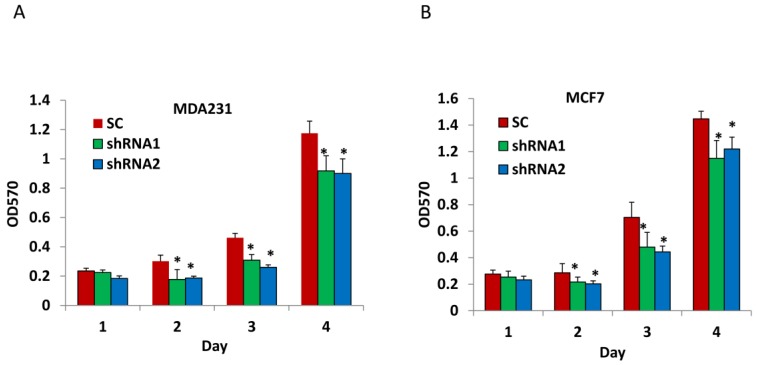
Silencing CLDN1 inhibits proliferation of breast cancer cells. (**A**,**B**) The proliferation of MDA-MB-231 cells (**A**) and MCF7 (**B**) transduced with different CLDN1 lentiviral shRNAs and SC control were examined by MTT assays. Data were presented as mean ± SD from three independent experiments (* *p* < 0.05).

To examine the role of CLDN1 in breast cancer cells, we first silenced expression of CLDN1 in MDA-MB-231 and MCF7 cells using two lentiviral shRNA vectors which target different regions of CLDN1 gene. To determine the effect of CLDN1 knockdown on cell proliferation, we performed MTT assays on MDA-MB-231 and MCF7 cells transduced with lentiviral CLDN1 shRNAs over a four-day culture period. We found that silencing CLDN1 expression using two different shRNAs significantly reduced cell proliferation when compared to SC transduced control cells in MDA-MB-231 and MCF7 breast cancer cells (Figure 2A,B).

### 3.3. Silencing CLDN1 Inhibits Clonogenicity of Breast Cancer Cells

**Figure 3:**
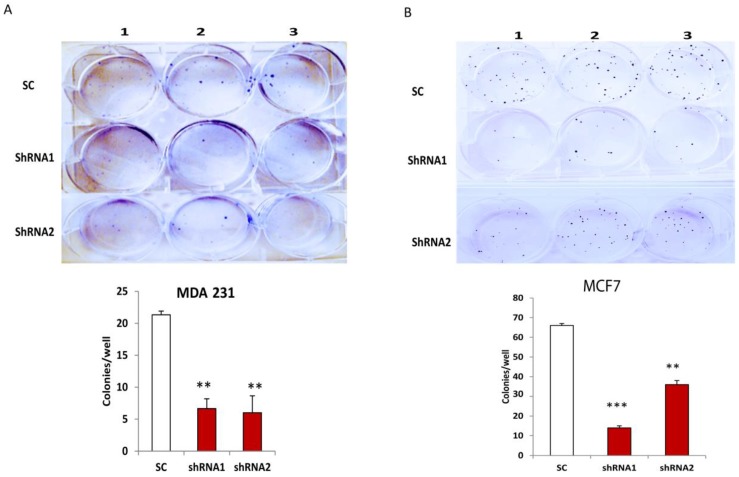
Silencing CLDN1 inhibits breast cancer cell survival. (**A**,**B**) Cell survival in MDA-MB-231 (**A**) and MCF7 (**B**) cells transduced with different CLDN1 lentiviral shRNAs and control vectors were examined by cell colony formation assays. Cell colonies were counted after culture (two weeks) in six-well plates and Crystal Violet staining. The number of colonies in CLDN1 lentiviral shRNA transduced cells was compared to that in control cells. Data were analyzed and presented from three independent experiments (** *p* < 0.01; *** *p* < 0.001).

To examine whether CLDN1 affected breast cancer cell survival, we performed colony formation assays in both MDA-MB-231 and MCF7 cells transduced with CLDN1 lentiviral shRNAs and SC control vectors. Cell colonies were counted following a two-week culture period. Silencing CLDN1 significantly reduced cell survival in both MDA-MB-231 (Figure 3A) and MCF7 (Figure 3B) cells transduced with CLDN1 lentiviral shRNA vectors compared to SC transduced control cells.

### 3.4. Silencing CLDN1 Inhibits Breast Cancer Cell Migration and Invasion

**Figure 4:**
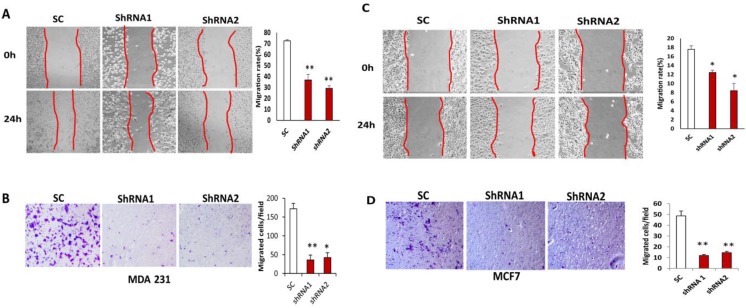
Silencing CLDN1 inhibits breast cancer cell migration. (**A**,**B**) Migration of MDA-MB-231 cells transduced with two different CLDN1 lentiviral shRNAs was compared with SC controls using wound healing (**A**) and Transwell migration assays (**B**), respectively. Data were presented from three independent experiments (* *p* < 0.05; ** *p* < 0.01). (**C**,**D)** Migration of MCF7 cells transduced with two different CLDN1 lentiviral shRNAss was compared with SC control cells using the wound healing (**C**) and transwell migration assay (**D**), respectively. (* *p* < 0.05; ** *p* < 0.01).

**Figure 5:**
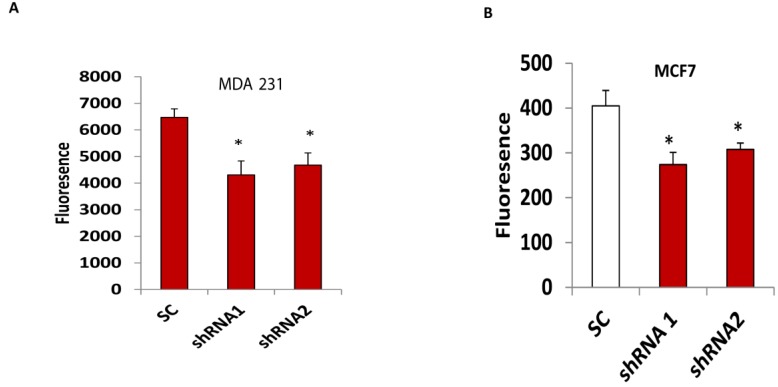
Silencing CLDN1 inhibits breast cancer cell invasion. (**A**,**B**) Invasion of two CLDN1 lentiviral shRNA transduced MDA-MB-231 (**A**) and MCF7 (**B**) cells were compared with SC transduced controls. Data were presented from three independent experiments (* *p* < 0.05).

To determine the role of CLDN1 in breast cancer cell migration, we performed wound healing assays on MDA-MB-231 and MCF7 cells transduced with different CLDN1 lentiviral shRNAs and SC control vectors. Breast cancer cell migration was significantly reduced in cell lines transduced with either CLDN1 lentiviral shRNA vector as compared to cells transduced with SC control following scratching and 24 h culture (Figure 4A,C). We also performed migration assay using transwell plates in MDA-MB-231 and MCF7 cells transduced with CLDN1 shRNAs and SC controls. As shown in Figure 4B,D, cell migration was significantly reduced in both shRNA transduced MDA-MB-231 or MCF7 cells when compared to SC transduced control cells. To examine how CLDN1 affects cell invasion, both CLDN1 lentiviral shRNAs and SC transduced MDA-MB-231 and MCF7 cells were seeded onto transwells coated with matrigels. Invaded cells were significantly reduced in both CLDN1 shRNA transduced MDA-MB-231 and MCF7 cells compared to SC transduced control cells, respectively (Figure 5A,B).

### 3.5. Silencing CLDN1 Inhibits EMT in Breast Cancer Cells

**Figure 6:**
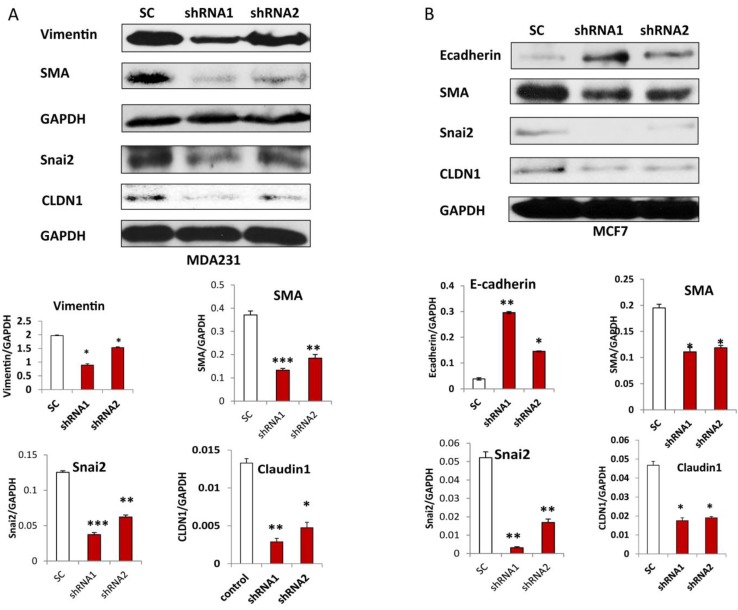
Silencing CLDN1 inhibits EMT in breast cancer cells. (**A**,**B**) EMT marker gene expression was detected in MDA-MB-231 and MCF7 cells transduced with CLDN1 shRNA and SC controls by Western blot, respectively. A representative Western blot was presented from three independent experiments. (* *p* < 0.05; ** *p* < 0.01; *** *p* < 0.001).

To examine whether CLDN1 expression regulates EMT in breast cancer cells, we examined the expression of the epithelial cell marker, E-cadherin, and the mesenchymal markers, Vimentin, Snai2 and SMA, in both MDA-MB-231 and MCF7 stable cells transduced with two different CLDN1 lentiviral shRNA and SC control vectors. The expression of E-cadherin was significantly upregulated, whereas expression of SMA and Snai2 was significantly downregulated in both CLDN1 shRNA transduced MDA-MB-231 and MCF7 cell lines when compared to SC controls. Both CLDN1 shRNAs were efficient in silencing CLDN1expression compared to scramble control (SC) transduced control cells as determined by Western blot (Figure 6A,B). However, Vimentin was also downregulated in MDA-MB-231 transduced cells whereas vimentin expression was not detectable in MCF7 cells.

## 4. Discussion

We have shown that CLDN1 was amplified and upregulated in the invasive breast carcinoma. There are five subtypes of breast cancer including luminal A, luminal B, Her2 overexpressing, Basa-like and Normal-Like [35]. CLDN1 expression was previously found to be relatively low in triple negative breast cancer [22], but was highly expressed in BRCA1-related breast cancer [36] and high-grade basal-like breast cancer [37,38]. Low expression of CLDN1 was associated with epigenetic regulation in estrogen positive breast cancer [39]. Taken together, these studies indicated that CLDN1 may play a dual role and can function as a tumor suppressor or oncogene depending on the breast cancer subtype [35]. Although there was no significant correlation between overall or disease-free survival and alteration of CLDN1 as analyzed from TCGA dataset, CLDN1 expression was correlated with the recurrence status and malignant potential of breast cancer as well as short disease-free interval [21]. It is still not clear whether CLDN1 expression is correlated with different grades or stages of breast cancer. Further investigation is required to elucidate the role of CLDN1 with the development of breast cancer.

We investigated the role of CLDN1 by examining two subtypes of breast cancer using a luminal A subtype of breast cancer cell line MCF7, which was positive for estrogen (ER) and progesterone receptors (PR), negative for Her2 and a basal-like subtype MDA-MB-231 cells, and negative for ER, PR and Her2. Silencing CLDN1 using lentiviral vector mediated RNA interference leads to reduced cell proliferation, migration, and invasion in breast cancer cell lines. Our results suggest that CLDN1 may be a therapeutic target in breast cancer metastasis. CLDN1 showed antiapoptotic effects in MCF7 breast cancer cells, while silencing CLDN1 promotes beta-catenin and E-cadherin expression [40]. Those results are consistent with what we found in MCF7 and MDA-MB-231 cells through highly efficient lentiviral vector mediated RNA interference. However, overexpression of CLDN1 in a luminal B subtype MDA-MB361 cells, which are positive for ER, PR and Her2, leads to cell apoptosis in 3D tumor spheroid cultures [41], inconsistent with the finding of the antiapoptotic effect of CLDN1 in MCF7 cell lines [24]. While this discrepancy between these studies is unclear, these findings may reflect a differential effect of CLDN1 in different cell types. Our studies indicated that CLDN1 demonstrated oncogenic properties in both MDA-MB-231 and MCF7 cells, although it was a tumor suppressor in MDA-MB-361 cells [40]. Those studies indicate that CLDN1 plays a distinct role in different breast cancer cells, which will require further study to understand how the ER, PR and her2 affect the functional outcome of CLDN1.

We showed that lentiviral vector mediated silencing of CLDN1 leads to inhibition of EMT in both MDA-MB-231 and MCF7 breast cancer cell lines. Although the mesenchymal marker vimentin was not detectable in MCF7 cells by Western blot, we observed high vimentin expression n MDA-MB-231 cells, suggesting that vimentin may at least contribute to a more invasive phenotype in MDA-MB-231 than in MCF7 cells. Previous studies demonstrated that Snai2 is a transcriptional repressor of CLDN1 in epithelial breast cancer cells [42]. However, we observed the downregulation of Snai2 following CLDN1 knockdown, suggesting that there is a potential negative feedback loop mechanism in controlling the interaction between Snai2 and CLDN1. High Snai2 levels reduced CLDN1 expression through transcriptional repression, which subsequently leads to reduced expression of Snai2 to maintain cellular hemostasis. However, it is still not clear how CLDN1 regulates EMT marker gene expression, including E-cadherin, Snai2, SMA, and vimentin. Interestingly, miRNAs have been shown to play a role in regulating CLDN1. For example, miR-155 functions as a tumor suppressor by targeting CLDN1 in ovarian cancer [17]. Therefore, miRNAs are also potential regulators in contributing EMT by targeting CLDN1, which may be an important area for further investigation.

Silencing CLDN1 inhibits EMT in both MDA-MB-231 and MCF7 breast cancer cells, indicating that CLDN1 is associated with tumor metastasis. Silencing CLDN1 expression may potentially inhibit breast cancer metastasis. In future studies, we will examine the effect of CLDN1 expression in orthotopic breast cancer xenograft mouse model by injecting CLDN1 stable cell lines generated using lentiviral vector mediated RNA interference. Moreover, lentiviral vector mediated RNA interference can be improved by thermotherapy, suggesting an improved efficacy of gene therapy in cancer treatment [43]. Furthermore, the recently developed lentiviral CRSIPR/cas9 (clustered regularly interspaced short palindromic repeats) system also provides a new approach in gene knockout by disrupting oncogene expression through gene editing. We have found that CRISPR/cas9 can efficiently silence the CLDN1 expression in breast cancer cell lines.

Our study has provided experimental evidence that lentiviral vector mediated RNA interference can efficiently silence CLDN1 expression in breast cancer cells, and silencing CLDN1 expression leads to reduced cell proliferation, migration, and invasion as well as inhibiting EMT. Our finding suggests that CLDN1 may be an important therapeutic target in breast cancer.
